# Acoustic prominence: Understanding speech patterns in children with Down syndrome

**DOI:** 10.1121/10.0039045

**Published:** 2025-08-01

**Authors:** Delin Deng, Miriam Lense, Stephen Camarata, Julie Mazzone, Doriana Lacitignola, Duane Watson

**Affiliations:** 1 Department of Psychology and Human Development, Vanderbilt University, Nashville, Tennessee 37235, USA; 2 Vanderbilt University Medical Center, Vanderbilt University, Nashville, Tennessee 37235, USA

## Abstract

Prominence in speech refers to the emphasis placed on certain words or syllables in English, making their salience through increased duration, increased intensity, and sometimes *f*0 dynamics. By marking acoustic prominence, speakers distinguish new from given information in speech. While a large body of relevant literature exists on how acoustic prominence is acoustically realized in typically developing (TD) children, little is known about how children with Down syndrome (DS) mark it. In this study, a “Puppet Show” task was conducted with 22 TD children and ten children with DS, one of whom was excluded because they were unable to produce the required target utterances despite multiple attempts, to compare the acoustic parameters used when distinguishing new and given information, focusing on mean *f*0, mean intensity, duration, and time-normalized *f*0. By using linear mixed-effects models and generalized additive mixed models, we demonstrated that children with DS have significantly higher overall mean *f*0 and lower intensity compared to TD children. The duration was not significantly different between the two groups. Our analysis further revealed distinct *f*0 contour patterns in the speech of DS children. While TD children exhibited clear rising and falling *f*0 contours to signal new and given information, DS children showed less controlled *f*0 contour patterns.

## I. INTRODUCTION

Acoustic prominence involves the use of specific acoustic features to highlight or emphasize elements of speech. Acoustic features, such as *f*0, intensity, duration, and *f*0 dynamics, play a significant role in making certain parts of speech salient to the listener in English. In typical speech development, these features are used proficiently by English-speaking children to convey meaning and indicate focus. Even at an early age, typically developing (TD) children adjust variations in *f*0, intensity, and duration to mark new information and emphasize important elements in their speech (see, for example, Crystal, 1986; Ito, 2014; Wonnacott and Watson, 2008).

While the acoustic marking of prominence has been well-studied in TD English-speaking children, far less is known about how it is realized in children with Down syndrome (DS), also known as Trisomy 21. DS is a genetic condition caused by the presence of an extra copy of chromosome 21 and is associated with a range of developmental differences, including intellectual disability, hypotonia (Latash et al., 2008), and motor coordination challenges. Critically, individuals with DS often experience pronounced speech and language difficulties, including delayed onset of speech, reduced intelligibility, and deficits in both segmental and suprasegmental aspects of speech production (Kent and Vorperian, 2013; Shriberg et al., 2001). Among families, speech clarity and communication effectiveness are frequently reported as the most pressing concerns for children with DS (Kumin, 2012).

Prior research on speech production in DS has largely focused on segmental difficulties, such as articulation errors and reduced speech intelligibility, and on general prosodic deficits, including monotonic intonation and reduced *f*0 variation (Kent and Vorperian, 2013; Shriberg et al., 2001). However, prosodic deficits, such as reduced *f*0 variation, flatter intonation contours, and impaired stress marking, also play a critical role in shaping speech intelligibility and listener perception in this population. These suprasegmental challenges may further impede pragmatic language use, which is already an area of vulnerability for many individuals with DS (Martin et al., 2009).

Moreover, existing studies on prosody in DS have primarily focused on scripted speech tasks or single-word productions, which do not fully capture the ways prominence is marked in natural discourse. Although previous research has documented atypical *f*0 patterns and reduced *f*0 variability in DS, the extent to which these acoustic features are modulated across different discourse conditions remains unknown. Given that prosodic features are essential for pragmatic communication and listener comprehension, investigating how children with DS adjust these features in different speech contexts could provide key insights into their communicative abilities.

This study aims to fill this gap by examining how children with DS use *f*0, intensity, and duration to mark acoustic prominence across different discourse conditions and whether their patterns differ from TD peers. Specifically, this study investigates the following research questions:

1. How do children with DS and TD children differ in their use of *f*0, intensity, and duration when marking acoustic prominence in speech across different discourse conditions?
2. Do children with DS show group-level *f*0 contour patterns that differ systematically from those of their TD peers across conditions?

## II. LITERATURE REVIEW

Marking acoustic prominence in speech refers to the use of various acoustic features to highlight or emphasize certain elements, making them salient to the listener. In English, it is essential for conveying meaning, indicating focus, and enhancing communication effectiveness. Acoustic features that contribute to prominence include *f*0, intensity, duration, and *f*0 dynamics (see, for example, Beckman, 1986; Beckman and Edwards, 1994; Cole et al., 2007; Cole et al., 2010; Kochanski et al., 2005; Ladd, 1996).

Typically, an increase in *f*0 can signal emphasis, new information, or focus in speech. While *f*0 is an important indicator of prominence, it may not always be the dominant feature. Intensity and duration often play more important roles in signaling prominence compared to *f*0 alone (Kochanski et al., 2005). This suggests a complex interplay between different acoustic features in establishing prominence. For instance, even young children as early as 4 yrs old begin to use rising intonation patterns to mark questions, signaling new information, though they may rely more on increased syllable duration at this stage (Patel and Grigos, 2006). Wonnacott and Watson (2008) investigated the use of acoustic prominence in 4-yr-olds and found that children use higher *f*0 and increased intensity on subject nouns that had shifted in discourse. This suggests that by age 4, children are already sensitive to discourse distinctions and can use acoustic prominence in ways similar to adults to signal new or shifted information in discourse (Buxo-Lugó et al., 2018).

In contrast, individuals with DS exhibit notable differences in their overall *f*0 use. Studies have shown that, even though *f*0 is often perceived as lower in individuals with DS, acoustic measurements demonstrate higher *f*0 values in speakers with DS, indicating a generally higher *f*0 in their speech (see, for example, Rochet-Capellan and Dohen, 2015; Seifpanahi et al., 2011). This higher *f*0 is coupled with increased variability, suggesting challenges in *f*0 control and stability (Rochet-Capellan and Dohen, 2015). These characteristics may be influenced by anatomical factors, such as hypotonia (Latash et al., 2008) and maxillary hypoplasia, which affect vocal fold function and control (Moura et al., 2008).

As for intensity, TD children increase the intensity of their speech when presenting new information (Wonnacott and Watson, 2008). This increase in intensity serves to draw attention to the more critical parts of their message (Ramos et al., 2020). As mentioned earlier, the integration of intensity with other features, such as *f*0 and duration, becomes more pronounced as children grow older and their speech skills mature (Kallay et al., 2022). In contrast, acoustic measurements indicate reduced intensity in overall speech production in children with DS (Pentz, 1987). This reduced intensity may be attributed to decreased respiratory support and hypotonia, which together reduce subglottal pressure and the motor coordination necessary for effective speech (Latash et al., 2008; Kumin, 2012). Respiratory function plays a crucial role in prosodic modulation by enabling variation in *f*0 and intensity (Kent and Netsell, 1971; Hoit and Hixon, 1987). In children with DS, multiple studies have reported respiratory challenges including upper airway obstruction, hypoventilation, and reduced tidal volume, all of which can limit vocal intensity and speech modulation (Pandit and Fitzgerald, 2012).

Regarding duration, studies suggest that TD children increase the duration of syllables to emphasize new information. Studies have shown that the ability to modulate duration develops over time, with older children displaying more adult-like patterns. For example, 7-yr-olds use both duration and *f*0 effectively to mark new information, whereas younger children might rely predominantly on duration (Patel and Grigos, 2006). Previous studies on the overall speech production in children with DS revealed that compared to TD children, children with DS tend to have longer and more variable duration (see, for example, Lynch et al., 1995; Brown-sweeney and Smith, 1997; Hohoff et al., 1998). This extended duration may be due to slower articulatory movements and motor control deficits associated with DS (Corrales-Astorgano et al., 2018). The prolonged duration may also result from a compensatory strategy to ensure intelligibility despite articulatory challenges.

Concerning *f*0 dynamics, in TD children, the use of rising and falling *f*0 contours helps differentiate between types of sentences. For example, declarative sentences typically end with a falling *f*0, while questions often end with a rising *f*0, signaling new information to the listener (Allen and Arndorfer, 2000). This control over *f*0 dynamics improves with age, becoming more sophisticated and varied. Children with DS, in contrast, have less control over the *f*0 dynamics. They often exhibit intonation patterns with reduced *f*0 range and reduced dynamics (Lee et al., 2009), which affects the naturalness and expressiveness of their speech (Lind et al., 1970). This lack of *f*0 variation potentially makes it more challenging for listeners to discern the relative importance of information within their speech.

As seen in the literature, there are notable differences in how these features are used between children with DS and TD children in their speech production. TD children demonstrate a more refined and integrated use of these features as they age, reflecting the maturation of multiple interacting systems. Anatomically, the elongation of the vocal tract and refinement of articulatory structures support more controlled modulation of *f*0 and intensity. Physiologically, improvements in respiratory–laryngeal coordination enable better control over breath support, subglottal pressure, and vocal fold vibration, which are necessary for dynamic prosody. Cognitively, the development of attention, working memory, and syntactic–pragmatic understanding supports more context-sensitive prosodic adjustments (Kent and Vorperian, 2013; Latash et al., 2008; Chapman and Hesketh, 2000).

In contrast, children with DS demonstrate distinct prosodic patterns that are influenced by atypical development in each of these domains. Anatomical differences, such as a smaller oral cavity and altered craniofacial structure, combined with generalized hypotonia, can impair articulatory precision and vocal control. Respiratory challenges including reduced tidal volume and inefficient breath support may further constrain intensity and pitch modulation (Pandit and Fitzgerald, 2012). On the cognitive level, limitations in working memory and processing speed may reduce the ability to integrate prosody with discourse context or to apply prosodic modulation flexibly across utterances. These interrelated factors contribute to reduced *f*0 variability, flattened intonation, and extended durations, all of which impact the clarity and communicative efficiency of speech in DS.

Recent studies have expanded our understanding of prosodic differences in DS using more ecologically valid and narrative-based tasks. For example, Zampini et al. (2016) found that children with DS exhibited less differentiated prosodic patterns than age-matched TD peers, even when controlling for language level. Zanchi et al. (2020) showed that prosodic anomalies persisted during narrative production and were particularly evident in sentence-final pitch contours. O’Leary et al. (2020) further confirmed that children with DS produce flatter prosody even in controlled single-word tasks, aligning with earlier findings of reduced intonation range. Corrales-Astorgano et al. (2018) extended these observations to adults, suggesting persistent prosodic atypicalities across the lifespan. Most comprehensively, a recent meta-analysis by Loveall et al. (2021) highlighted that individuals with DS consistently exhibit reduced pitch variation and slower speech rate compared to TD individuals, across a range of task types. These studies highlight the need to explore prosody in DS using context-sensitive paradigms that go beyond scripted production.

While prior studies have provided important insights into prosodic patterns in DS, including some based on connected speech or narratives (e.g., Zampini et al., 2016; Zanchi et al., 2020), many focused on global measures or used elicitation tasks with limited contextual variation. Our study builds upon this work by using a semi-structured, discourse-sensitive paradigm, the “Puppet Show” task, that systematically manipulates the discourse status of referents. While this task does not constitute fully spontaneous or interactive conversation, it does simulate communicative demands by requiring participants to adapt utterances based on preceding context. This design allows us to examine how children with DS combine acoustic features, such as *f*0, intensity, duration, and *f*0 dynamics in response to different discourse conditions. In doing so, we move beyond isolated word-level assessments to analyze prosody in contextually grounded utterances, offering a more detailed view of prominence marking strategies compared to TD peers.

## III. METHODS

### A. Participants

Twenty-two TD children (eight male, 14 female) and a larger pool of children with DS were initially recruited for this study. Ten children with DS (five male, five female) were formally enrolled, but only nine were included in the final analysis. One participant (DS08) completed the experimental protocol but was unable to produce target utterances despite multiple attempts. In the TD group, one participant exhibited a higher rate of disfluencies than others, but their data were retained as the disfluencies did not interfere with the acoustic realization of the target words.

An additional three children with DS (DS11–DS13), who were among the youngest in the sample, participated in preliminary screening but did not proceed to this task. These participants appeared not to fully understand the task demands, and testing was discontinued by the clinician to minimize participant burden. Participant identification numbers (IDs) are based on the original recruitment order and have been preserved in anonymized form for consistency across records.

The inclusion criteria were: (a) chronological age between 3 and 6 yrs for TD group and between 4 and 16 yrs for the group with DS, (b) regular production of utterances of two to three–word (confirmed via parent report during screening and subsequent assessments), (c) typical development of speech and language (TD group only), (d) passing a hearing screening, and (e) living in a home where English is the primary language. To assess (b), caregivers completed a brief intake questionnaire describing their child’s typical communication behaviors, including whether they used spoken language and how many words they typically combined per utterance (e.g., single words, short phrases, or full sentences). This information was clarified during a Zoom (Zoom Video Communications, Inc., San Jose, CA) consent call. Additionally, a trained clinician calculated each participant’s mean length of utterance (MLU) during a baseline free-play language sample to ensure that only children who consistently produced two- to three-word utterances were included. For criterion (d), hearing ability was assessed based on caregiver report during the initial intake process. Although no formal audiometric testing was conducted, the clinician monitored participant behavior during all sessions and confirmed that none of the children showed signs of hearing difficulty (e.g., failure to respond to auditory prompts, repeated requests for clarification). No child was excluded due to suspected hearing impairment. The exclusion criteria were: (a) uncontrolled seizures, (b) diagnosed attention-deficit/hyperactivity disorder (ADHD) or autism, (c) apraxia secondary to a diagnosed neurological disorder, (d) developmental disorders (TD group only), (e) stuttering, and (f) severely disruptive behavior as reported by the parent. Participants were compensated for their time with enrichment sessions.

Written informed consent was obtained from the parent or legal guardian of each participant in accordance with the approved institutional ethics protocol. In addition, verbal assent was obtained from each child prior to beginning the task. A trained clinician read an assent script written in simplified, developmental-appropriate language, explaining the purpose of the study, what would happen during participation, and the child’s right to withdraw at any time. Participants were required to verbally indicate their agreement before proceeding. The clinician monitored participants throughout the session for signs of fatigue or distress and offered breaks as needed. These procedures ensured informed and voluntary participation from both the DS and TD groups. Table I provides the detailed demographic characteristics of DS participants:

To ensure comparability across groups, inclusion criteria for the DS group focused on expressive language ability rather than chronological age. Participants were required to produce multi-word utterances (two to three words per utterance), as confirmed through parental reports and screening assessments. As shown in Table I, the DS group in this study included participants ranging from 8 yrs and 9 months to 15 yrs of age (*M* = 12.35 ± 2.16), covering a broader developmental span than the TD group, whose ages fell between 3 and 6 yrs (*M* = 4.84 ± 0.89). Although the DS group was significantly older in chronological age, their age-equivalent speech abilities, based on the WCAB test score, ranged from younger than 2 yrs to approximately 7 yrs and 4 months, aligning closely with the expected linguistic abilities of the TD participants. Prior research suggests that speech and language development in individuals with DS follows a delayed but protracted trajectory, with older children often demonstrating speech characteristics similar to much younger TD peers (Chapman and Hesketh, 2000). The inclusion of a wider age range was necessary to capture a representative range of speech abilities within the DS population while ensuring comparability with the younger TD participants in terms of linguistic functioning. Restricting the DS group to a narrower age range would have likely excluded participants whose speech abilities were most relevant to the comparison with younger TD children. While intellectual disabilities are a defining characteristic of DS, speech and prosodic development do not always correlate directly with cognitive ability, making linguistic output a more appropriate matching factor (Eadie et al., 2018).

Although maturational differences, particularly those associated with puberty, may have influenced acoustic characteristics in the older DS participants, previous research suggests that prosodic deficits in DS persist throughout childhood and adolescence, independent of chronological age (Moura et al., 2008; Rochet-Capellan and Dohen, 2015). Additionally, statistical approaches were implemented to account for individual variability, and mean *f*0 values were normalized within participants to minimize the impact of age-related vocal changes. While we acknowledge that including a wider age range introduces variability, it also provides a more ecologically valid representation of speech characteristics in DS. This approach reflects the reality that speech and prosodic development in DS progresses along a more gradual and extended timeline, with older children continuing to refine aspects of their prosody at stages when TD children have typically already mastered these features.

While this group size imbalance may appear substantial, it reflects the practical challenges of recruiting children with DS for acoustic research, including lower prevalence, cognitive variability, and task compliance issues. Nevertheless, the current sample provides valuable insights when analyzed with robust statistical methods. To enhance transparency and enable speaker-specific interpretation, we provide a supplementary table (Table S1) reporting individual-level acoustic and demographic data for all TD and DS participants, including mean, standard error (SE), minimum (min), and maximum (max) trials attempted and valid. To supplement the group-level comparisons, we provide speaker-specific visualizations of *f*0 contours for each participant with DS in supplementary material Fig. S1. These plots illustrate dynamic variation across the three discourse conditions, highlighting the heterogeneity of prosodic modulation strategies within the DS group. These supplementary material supports both the interpretation of current findings and future meta-analytic work.

### B. Experiment

Sentences were elicited through a “Puppet Show” task, an interactive speech elicitation method that has been previously used to investigate acoustic prominence in children’s speech (Wonnacott and Watson, 2008). This task was designed to elicit spontaneous yet structured speech, ensuring that children engaged in natural discourse while producing controlled target utterances. The experimenter used animal puppets to perform a series of scripted actions, following a structured but flexible format tailored to the child’s developmental level.

The task involved five puppets performing three different actions (*hitting, kissing, and hugging*). Each trial consisted of two consecutive scenes, with two puppets interacting in each. In each scene, one puppet acted as the agent while the other served as the recipient (e.g., “*The cow kissed the frog*.”, “*The duck hugged the donkey*.”). The first scene functioned as the context scene, setting the discourse conditions for the target utterance in the second scene. The target words of interest were always the actors in the second (target) scene (see Sec. III C).

Following each scene, the experimenter prompted the child to describe the event (e.g., after the first scene: “*What happened*?”; after the second scene: “*And then what happened*?”). Some participants required prompting or scene repetitions to produce an appropriate response. If the participant was still unable to respond as expected, the experimenter modeled the response with neutral prosody, and the child was asked to imitate the phrase.

This task was adapted from Wonnacott and Watson (2008), who used a similar discourse-based paradigm to examine acoustic prominence in young childrens’ speech. The decision to use this approach rather than single-word production or scripted sentence repetition tasks was motivated by its ability to capture natural discourse-driven prosodic variation. Unlike previous studies focusing on intonation deficits in DS within highly controlled speech conditions, this approach allows for a more ecologically valid assessment of how children with DS and TD peers use *f*0, intensity, and duration to mark prominence in connected speech.

### C. Tokens

Each participant was asked to produce 18 sentences, involving five different animals and three actions, with each action repeated twice in a row (For example: “*The cow hit the donkey*.” [followed by] “*The dog hit the frog*.”). All participants produced the sentences in the same order, without randomization or counterbalancing of lists. All utterances were labeled by trained research assistants (RAs) for discourse role (context vs target) and number of attempts per item. RAs were blind to study hypotheses and were not involved in data collection. For analysis, we included only first-attempt target utterances to ensure consistency in participant task engagement and to minimize the influence of practice, fatigue, or prompting.

All selected utterances were reviewed for acoustic quality. Trials were excluded if they contained disfluencies, background noise, or production errors that compromised segmental clarity or made pitch tracking unreliable (e.g., due to creaky voice or glottalization). Acoustic preprocessing was conducted manually to ensure accurate extraction of pitch, intensity, and duration. After applying these criteria, a total of 593 valid target tokens were included in the final dataset (245 for the DS group; 348 for the TD group). The first author (D.D.) then annotated these occurrences for information status.

The study had three conditions [(a) is the context sentence and (b) is the target sentence]:

1. New condition:
  - The **cow** hit the donkey.
  - The **dog** hit the frog.
  In this condition, the target sentence differs from the first in both subject (agent) and object (recipient) positions. Entirely new information is introduced. These sentences are expected to elicit greater prominence, as speakers often use higher *f*0, increased intensity, and longer duration to highlight new or important information.
2. Given-shift condition:
  - The duck hugged the **cow**.
  - The **cow** hugged the dog.
  Here, the subject of the target sentence was previously mentioned as the object of the context sentence. This shift in position, while maintaining a familiar subject, can sometimes trigger an accent that signals contrast. These sentences were expected to elicit higher *f*0, increased intensity, and longer duration, though potentially less so than in the “new” condition, where entirely new information may trigger stronger prominence marking.
3. Given-nonshift condition:
  - The **duck** kissed the frog.
  - The **duck** kissed the donkey.
  In this condition, the subject of the target sentence was previously mentioned in the context sentence and remained in the same position in the target sentence. These sentences were expected to show the least prominence, as they involve repetition of known information, which usually receives less acoustic emphasis in natural speech.

Target words were selected based on their discourse role in the Puppet Show task (i.e., as agents in the second sentence of each trial). No phonological constraints (e.g., presence or absence of voiceless segments) were imposed in selecting these items. Steps taken to ensure accurate *f*0 measurement despite this variation are described below.

### D. Acoustic measurements

Recordings for both groups were made using high-quality digital recorders [Marantz PMD661 (Marantz Professional, Cumberland, RI) for DS; Zoom H6 Handy Recorder (Zoom Corporation, Hauppauge, NY) for TD], with the microphone placed at the center of the table, approximately 15 in. from the participant’s mouth. In all sessions, the microphone was equidistant from the participant and the experimenter. The same room, table, and chair configuration were used across sessions to ensure consistency in recording conditions and intensity measurements. Annotation boundaries were manually adjusted based on the spectral features of each word. Acoustic measures, including mean *f*0, mean intensity, mean duration, and time-normalized *f*0, were extracted using ProsodyPro (Xu, 2013). To ensure the reliability of *f*0 values, especially in the presence of potentially perturbing voiceless segments, *f*0 tracks were visually inspected. Measurements were verified to align with voiced regions of the signal where pitch tracking is most stable. For each target word, ten time-normalized measurement points were extracted, and dynamic *f*0 values were converted into semitones using the formula *st* = 12 * *log*2(f*0/reference* f0) (Zahner-Ritter et al., 2022). Reference values of 210 Hz (male) and 227 Hz (female) were used, and only semitone values between −8 and 13 were included, resulting in 5720 of 5930 measurement points retained for analysis.

### E. Statistical analysis

Given the limited sample size of ten children with DS, a power analysis was conducted to assess the feasibility of detecting meaningful effects. Using PASS (NCSS, 2023) software, we estimated that with six predictors across two primary speech measures (accuracy and intelligibility), our analyses can detect correlation coefficients greater than 0.63 (or less than −0.63) with 0.80 power. Furthermore, an *R*^2^ change of 0.09 was estimated for one correlate while controlling for another of equal association strength, using a one-tailed test with 0.80 power. This is an adjusted estimate based on previous research, which found an *R*^2^ change of 0.18 for general cognitive delay and speech intelligibility in students with DS when the sample size was doubled (*N* = 20).

We acknowledge that the current sample size limits the ability to detect small effects and generalize findings. However, this study serves as an exploratory investigation into prosodic characteristics in DS, providing a foundational dataset for future larger-scale studies. While effect sizes in the current study may be larger due to sample constraints, the findings still contribute important insights into the speech patterns of an understudied population.

For statistical analysis, two models were used. First, linear mixed-effects regression models were constructed using the *lme4* package in *R* (Bates et al., 2015; R Core Team, 2024) to analyze the impact of and interaction between various factors on the three acoustic features (mean *f*0, mean intensity, and duration). All categorical variables were coded using deviation coding, and a single model was fit for each acoustic measure using the combined data from both participant groups. The model included fixed effects of group (TD vs DS) and condition (given_nonshift, given_shift, new), as well as random intercepts for participant and item (target word).

While *lme4* does not directly support modeling group-level variance differences (heteroskedasticity), we addressed this by including participant- and item-level random intercepts to capture speaker- and word-specific variability. Initially, more complex models with random slopes for the group × condition interaction were tested but failed to converge due to boundary fit issues, so we retained a simpler model structure with only random intercepts for stability. We complemented the inferential analyses with descriptive statistics and individual-level visualizations to avoid overinterpreting marginal effects.

The *R* formula for the final models is provided below:

lmer(meanf0∼group∗condition+(1|participant)+(1|item),data=data),lmer(mean_intensity∼group∗condition+(1|participant)+(1|item),data=data),lmer(duration∼group∗condition+(1|participant)+(1|item),data=data).

To explore time-varying *f*0 contours, we also applied generalized additive mixed models (GAMMs) (Wieling, 2018) using the *mgcv* package (Wood, 2011, 2017). GAMMs were well-suited to this analysis because they allow flexible modeling of non-linear trajectories while accounting for individual variability. The dependent variable was normalized *f*0, modeled over ten equidistant time points across the target word. Data were preprocessed by excluding disfluent or noisy tokens and manually verifying *f*0 tracking.

The final GAMMs included fixed effects for discourse condition and group, as well as several smooth terms to model non-linear *f*0 trajectories. Specifically, the model incorporated a smooth for time by condition [*s(time_scaled, by* = *condition)*], a smooth for time by group [*s(time_scaled, by* = *group)*], and a random smooth for each participant [*s(time_scaled, participant, bs* = “*fs*”, *m* = *1)*]. This structure allowed the model to capture dynamic variation in *f*0 contours over time, while accounting for both between-group patterns and individual speaker variability.

We used *bam()* for efficient fitting with autocorrelation adjustment (*rho* = 0.8, AR.*start* structure) and evaluated model fit by incrementally adding interactions and adjusting the number of base functions (Sôskuthy, 2021; Zahner-Ritter et al., 2022). Scaled *t*-distributions were used to reduce the influence of heavy-tailed residuals (van Rij et al., 2019).

The formula for the model is provided below:

bam(f0∼condition∗group)+s(time_scaled,by=condition)+s(time_scaled,by=group)+s(time_scaled,participant,bs=fs,m=1),AR.start=data$AR.start,rho=0.8,data=data,discrete=TRUE.

## IV. RESULTS

In this section, we first present descriptive statistics for key acoustic features, including mean *f*0, intensity, and duration, across the DS and TD groups and for the three discourse conditions (“given_nonshift,” “given_shift,” and “new”). These raw values provide an initial understanding of group differences. Following this, we apply normalization procedures and conduct linear mixed-effects models to account for individual variability and item-specific effects, allowing for a more refined analysis of the patterns in acoustic modulation across groups and conditions.

### A. Mean *f*0

Table II presents the raw mean *f*0 values and standard deviations for the DS and TD groups across the three discourse conditions (“given_nonshift,” “given_shift,” and “new”).

**FIG. 1.**
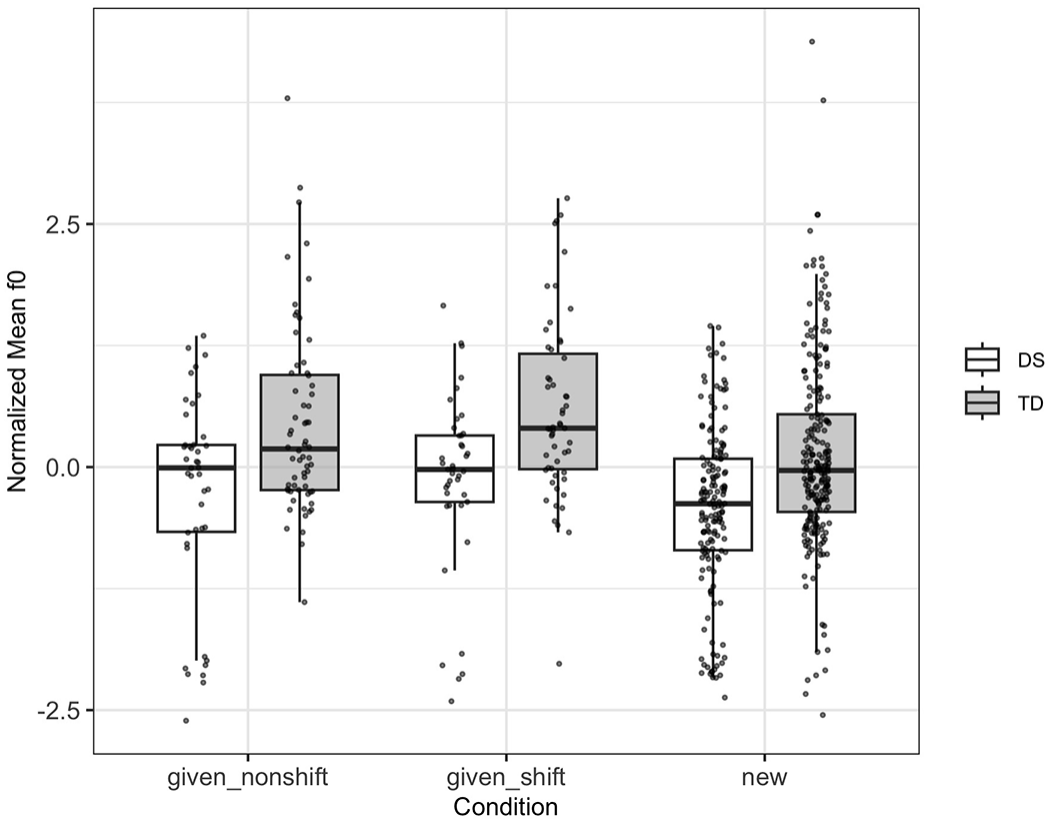
Boxplot showing the distribution of normalized mean *f*0 by discourse condition and participant group. Individual data points are plotted to highlight within- and between-group variability.

Figure 1 displays the normalized mean *f*0 values by condition and participant group. While this visualization provides an intuitive sense of between- and within-group variability, it is limited in its ability to account for individual baseline differences and repeated items.

Although the TD group exhibits slightly wider interquartile ranges and more dispersed data points, raw and normalized descriptives do not control for participant-level baselines or item-level effects. To address this, we applied linear mixed-effects modeling, which accounts for both fixed effects (group and condition) and random effects (participants and target items). This modeling strategy allows us to isolate the unique contributions of group and condition while controlling for nested data structure.

As shown in Table III, the model indicates that the TD group, after normalization, shows significantly lower mean *f*0 than the DS group (estimate = −0.29, *p* = 0.034), despite appearing higher in raw Hertz terms. This reversal highlights the need for normalization and modeling to control for age-related anatomical differences (e.g., shorter vocal tracts in younger TD children) and speaker-level variation. The “new” condition significantly increased *f*0 (estimate = 0.12, *p* = 0.031), suggesting discourse-driven modulation. However, interactions between group and condition were not significant.

### B. Mean intensity

Table IV presents the raw mean intensity values and standard deviations for each discourse condition and participant group. On average, children with DS produced higher absolute intensity values than their TD peers across all three conditions.

**FIG. 2.**
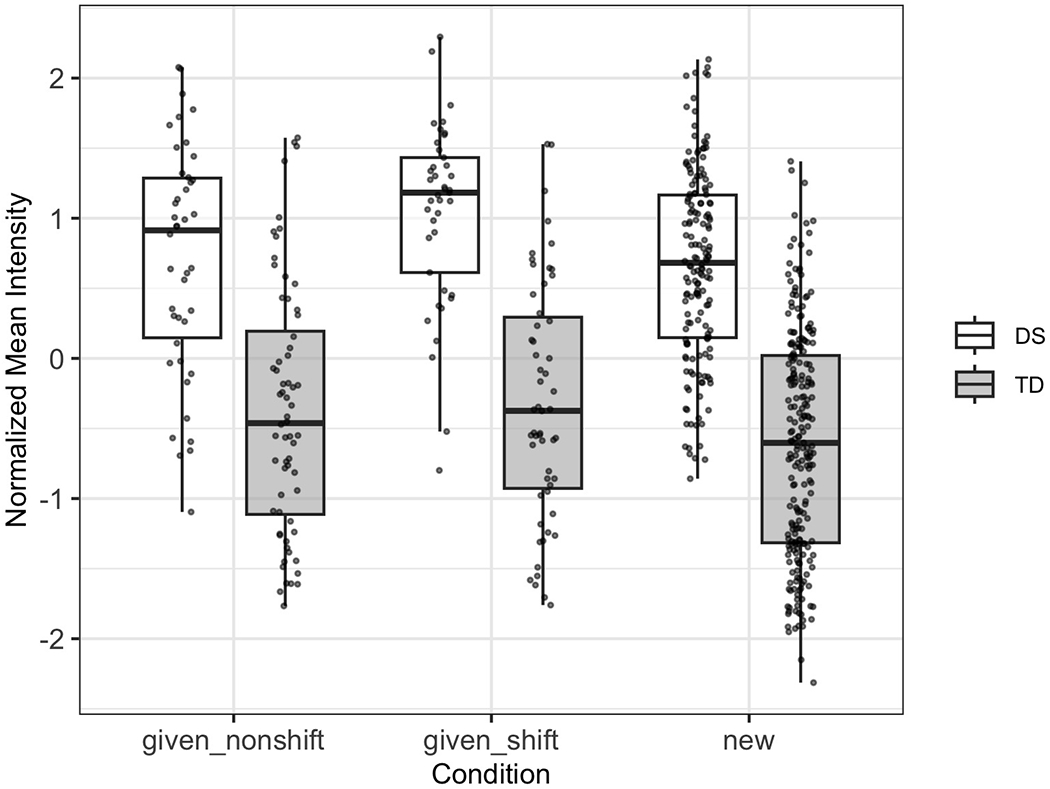
Boxplot showing the distribution of normalized mean intensity by discourse condition and participant group. Each dot represents an individual utterance.

Figure 2 shows the normalized mean intensity distributions across conditions for both groups. This visualization illustrates how intensity varies across discourse contexts within each group. A more detailed examination of condition and group effects is provided in the linear mixed-effects model results in Table V.

Table V presents the results of the linear mixed-effects model analysis for normalized mean intensity. The intercept, corresponding to the DS group in the “given_nonshift” condition, was not significant (estimate = 0.17, *p* = 0.224). There was a strong main effect of group: TD participants demonstrated significantly higher normalized mean intensity than the DS group [estimate = 0.63, 95% confidence intervals (CI) (0.37, 0.89), *p* < 0.001], indicating a reversal of the raw trend once speaker-level baseline variability was accounted for. The “new” condition also yielded significantly higher normalized intensity values than the “given_nonshift” baseline [estimate = 0.16, 95% CI (0.08, 0.24), *p* < 0.001], while the “given_shift” condition was not significantly different (*p* = 0.634). No interaction between group and condition reached significance, suggesting that both groups modulate intensity across discourse conditions in similar ways.

Although raw intensity values indicated that DS speakers were louder overall, the linear mixed-effects model revealed that this pattern reversed when controlling for individual variability, with TD speakers exhibiting higher normalized intensity. This suggests that the raw group difference may reflect broader individual or physiological factors, such as age or vocal effort, rather than systematic prosodic strategies. Importantly, the absence of a significant group-by-condition interaction indicates that both groups modulated intensity similarly across discourse contexts. Thus, while intensity may serve as a feature for discourse marking, the current results do not support the claim that DS children rely on this feature more actively or differently than their TD peers.

### C. Duration

Table VI presents the raw mean duration values and standard deviations by condition and group. On average, both groups showed increased durations in the “new” and “given_shift” conditions relative to “given_nonshift,” though the pattern differed in strength and variability across groups.

**FIG. 3.**
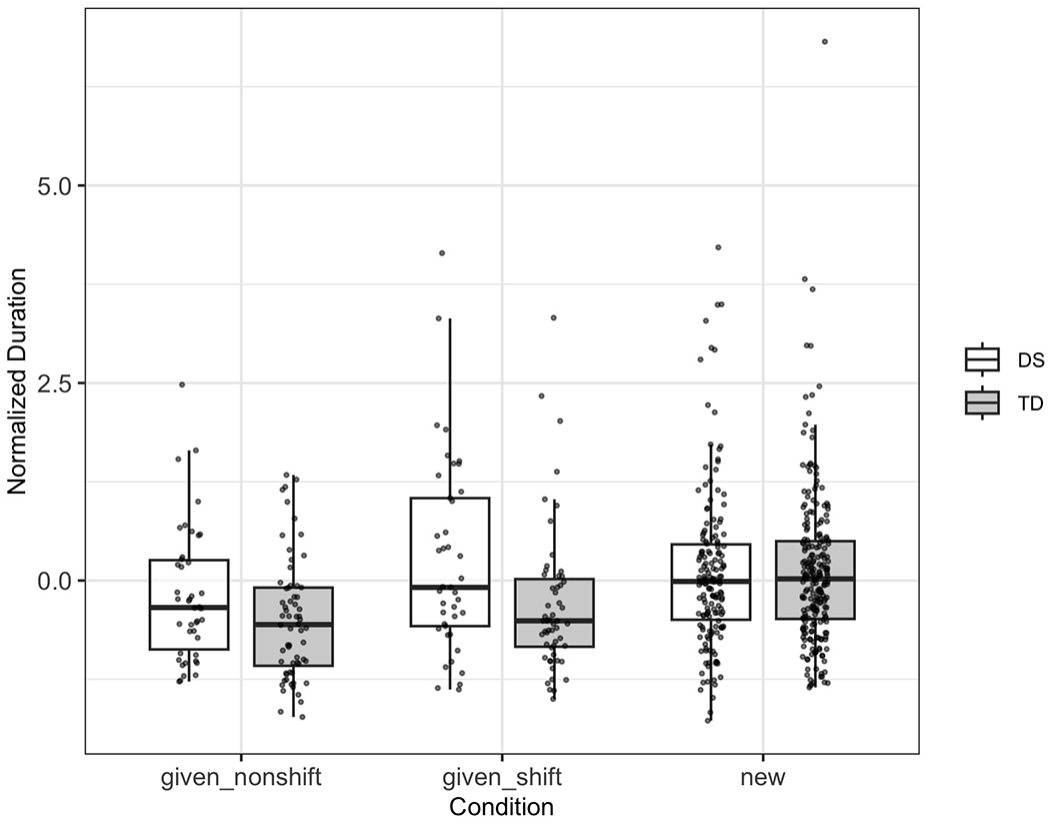
Boxplot showing the distribution of normalized duration by discourse condition and participant group. Individual data points are plotted to highlight within- and between-group variability.

Figure 3 displays the distribution of normalized duration across three conditions (“given_nonshift,” “given_shift,” and “new”) for the DS and TD groups.

Table VII summarizes the results of the mixed-effects analysis on normalized duration. The baseline intercept is not significant, nor is the main effect of group, suggesting no overall difference in normalized duration between TD and DS participants. The “given_shift” condition is associated with a small but significant reduction in duration (estimate = −0.15, *p* = 0.016).

A critical finding is the significant interaction between TD group and “new” condition (estimate = 0.13, *p* = 0.038). This result indicates that the TD group modulated their durations more distinctly in the “new” discourse context than the DS group. In contrast, there was no group difference in how duration was modulated in the “given_shift” condition.

These results suggest that although raw durations appear higher for the DS group overall, their pattern of modulation across discourse contexts is less pronounced. The TD group, in contrast, exhibited a more flexible adjustment of duration, particularly when marking new referents. This supports the interpretation that DS children may rely less on duration as a prosodic cue for information structure.

### D. GAMMs

**FIG. 4.**
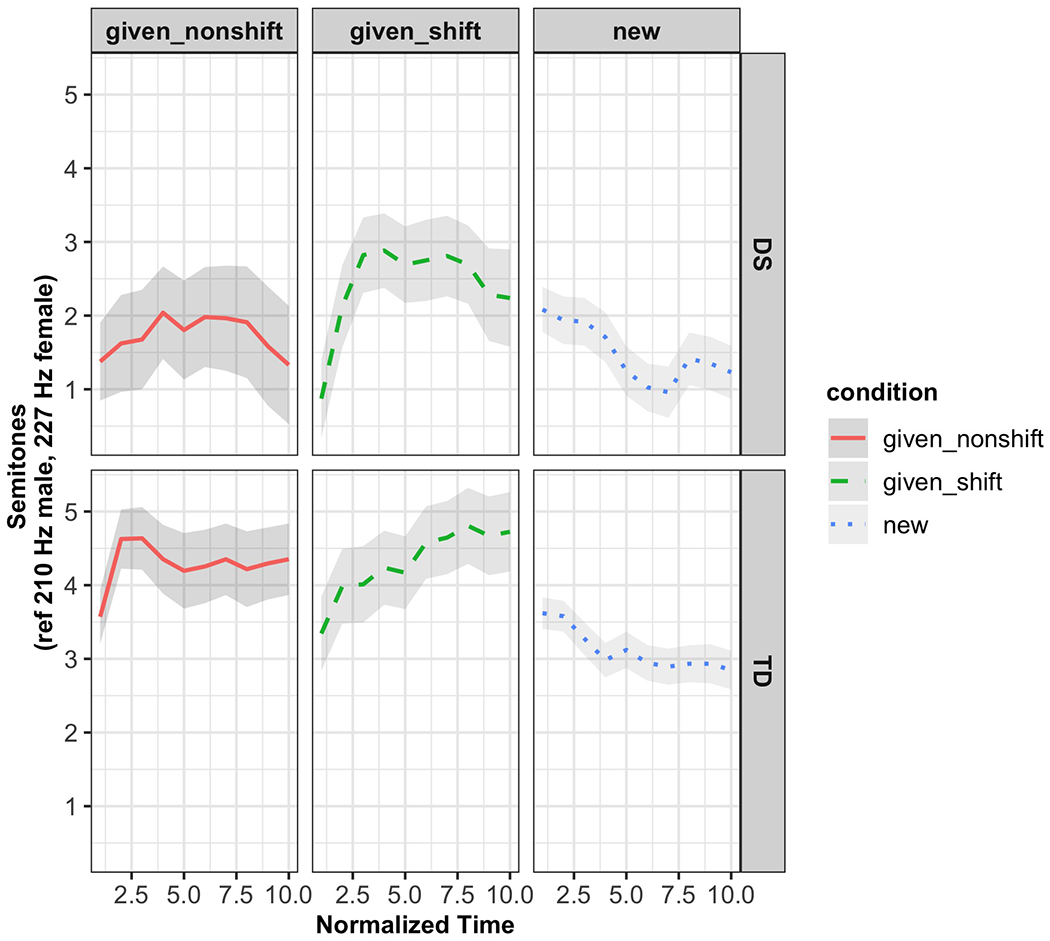
Time-normalized *f*0 contours for target words across discourse conditions and participant groups. Contours represent predicted values from GAMMs, with 95% CI shaded. Time 0-1 corresponds to the duration of the target word, normalized across ten equidistant bins. ref = reference *f*0 value (210Hz male, 227 Hz female).

Figure 4 presents the raw *f*0 contour patterns across different conditions for both the DS and TD groups. The y axis represents semitone values, normalized relative to reference frequencies (210 Hz for males and 227 Hz for females), while the *x* axis represents normalized time.

As shown in Fig. 4, in the “given_nonshift” condition, both groups show relatively flat contours, though the TD group maintains more consistent trajectories with narrower CI. The DS group, by contrast, displays wider intervals, particularly toward the end of the word, indicating greater intra-group variability in pitch modulation. In “given_shift” condition, the DS group shows a sharp initial rise followed by a rapid drop, while the TD group exhibits a steadier, more linear rise. In the “new” condition, both groups show an overall decline in *f*0, but the drop is steeper and more variable for the DS group. These broader confidence bands suggest less consistent control over pitch trajectory within the DS group, potentially due to articulatory or motor planning challenges.

**FIG. 5.**
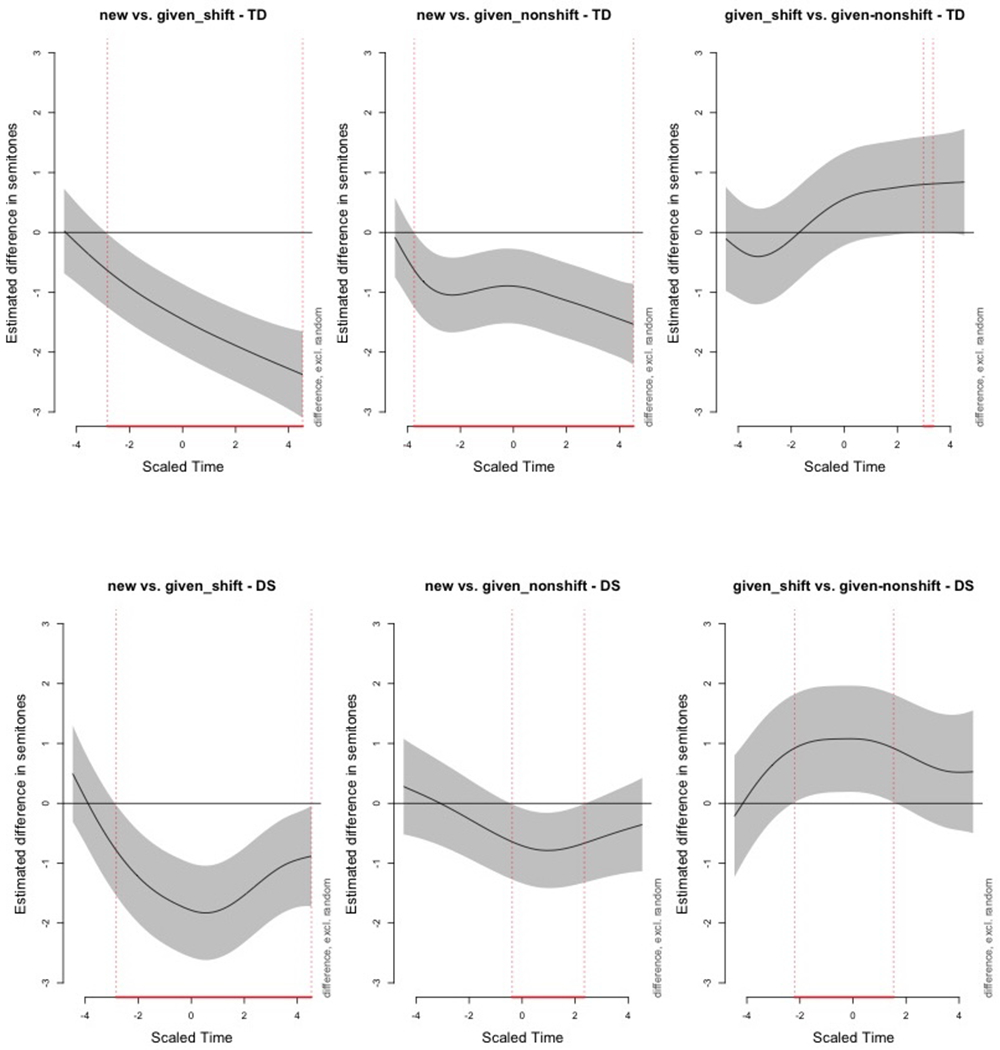
Estimated difference smooths from GAMMs comparing *f*0 contours between discourse conditions, shown separately for the TD (top row) and DS (bottom row) groups. Each panel displays the estimated *f*0 difference (in semitones) across scaled time, which spans ten equidistant points over the duration of the target word (from −5 to 5). Shaded regions represent 95% CI. Time regions where the confidence band does not include zero indicate statistically significant differences in *f*0 between conditions during that portion of the word.

To further isolate condition contrasts over time, Fig. 5 plots difference smooths derived from the GAMMs.

In the “new vs given_shift” contrast, both groups show early *f*0 decreases, with a stronger and more sustained drop in the DS group. This suggests sharper pitch modulation for new information among DS participants, possibly compensatory or exaggerated in nature. For “new vs given_nonshift,” the TD group exhibits a modest mid-word decrease in *f*0, whereas the DS group shows a noisier and shorter divergence, again highlighting greater inter- and intra-speaker variability. In “given_shift vs given_nonshift,” both groups show increasing contours toward the end of the word, but the DS group’s difference is larger and spans a longer portion of the word.

Table VIII summarizes model coefficients. Parametric effects reflect group-level *f*0 differences per condition, while smooth terms capture the nonlinear temporal contouring of pitch. For the DS group, time-dependent modulation of *f*0 was significant in “given_shift” and “new,” but not “given_nonshift.” For the TD group, all three conditions showed significant temporal effects, with the most pronounced contouring during “given_shift.”

GAMMs reveal that both groups modulate *f*0 dynamically, but the TD group does so systematically across conditions. Children with DS show greater variability and less consistent control, particularly in contexts requiring nuanced prosodic adjustments. The GAMMs approach captures these fine-grained differences by modeling pitch trajectories over time, beyond what mean-based summaries or static statistics can show.

## V. DISCUSSION

This study addressed two primary research questions:

1. Do children with DS differ from TD peers in their use of acoustic features, specifically *f*0, intensity, and duration, to mark information structure in speech?
2. Do children with DS exhibit distinctive patterns in the temporal modulation of *f*0 contours compared to TD children?

To address the first question, our findings from both descriptive and inferential analyses indicate that children in both groups modulate acoustic features based on discourse context. In particular, both DS (*N* = 9) and TD (*N* = 22) participants showed increased intensity in the “new” condition, consistent with prior literature demonstrating prosodic marking of informational novelty. However, mixed-effects models revealed that children with DS displayed significantly lower normalized intensity and less differentiated duration patterns than their TD peers, suggesting a reduced or less robust use of these cues for signaling prominence. While the “new” condition elicited increased intensity across groups, the interaction terms suggest TD children may be more consistent or effective in deploying this cue.

For mean *f*0, the raw descriptive statistics initially suggest higher *f*0 values in the TD group. However, this pattern reflects unnormalized acoustic measures, which are confounded by age-related anatomical differences such as shorter vocal tracts in younger TD children. After accounting for speaker-level variability through mixed-effects modeling, the direction of the effect reverses: children with DS exhibited significantly higher normalized mean *f*0 than TD peers. This aligns with prior findings attributing higher *f*0 in DS speech to atypical laryngeal structure and vocal fold control associated with hypotonia and craniofacial morphology. These findings demonstrate the necessity of statistical modeling for accurately capturing group-level trends when baseline variability is high.

To answer the second research question regarding *f*0 contour dynamics, GAMMs revealed that both groups modulated *f*0 over time in response to discourse conditions. However, TD children exhibited smoother, more predictable contour trajectories, especially in the “given_shift” context where discourse requires referent reactivation. In contrast, children with DS showed significant but less stable modulation across conditions. While GAMMs detected significant time effects for the DS group, particularly in “given_shift” and “new” contexts, the shapes of the contours were more variable across participants.

Although we do not report statistical metrics of intraspeaker variability directly, the more irregular *f*0 trajectories in the DS group, visible in Figs. 4 and 5, may suggest challenges with motor timing or prosodic planning. That said, we interpret these differences cautiously: variability patterns were not the primary analytic target of the GAMMs, and our model includes random smooths to account for speakerspecific trends.

These results suggest that children with DS demonstrate sensitivity to discourse context and can adjust acoustic features to mark prominence. However, their acoustic modulations, particularly of *f*0 over time, appear less refined or less consistent than those of their TD peers. This pattern may reflect combined effects of physiological constraints, motor planning challenges, and differences in linguistic–pragmatic processing. Future work should further isolate the contributions of these mechanisms and directly quantify within- and across-speaker variability to better understand the stability of prosodic control in DS speech.

## VI. CONCLUSION

This study provided a comprehensive analysis of acoustic prominence in the speech of 22 TD children and nine children with DS using a semi-structured “Puppet Show” elicitation task. We focused on how different discourse conditions (“new,” “given_shift,” and “given_nonshift”) influence the use of acoustic features, such as *f*0, intensity, duration, and *f*0 contour patterns, to mark acoustic prominence in speech.

By using LMER and GAMMs, we found evidence that children with DS exhibit distinct prosodic modulation patterns compared to TD children. Specifically, TD children showed more consistent and differentiated use of acoustic cues, such as intensity and duration in response to discourse context. While the descriptive statistics initially suggested higher mean *f*0 in TD children, the normalized and model-based analyses revealed no significant group-level elevation in *f*0 for DS children. These findings emphasize the importance of model-based inference in clarifying speakerlevel variability and discourse-driven prosodic patterns.

Nonetheless, some limitations warrant caution in interpretation. The relatively small and unmatched sample, particularly within the DS group, constrains generalizability. Additionally, variability noted in DS participants was visually observable in the contours and scattered but not statistically tested; future research should directly assess inter- and intra-speaker variability.

Future studies should expand on these findings by using larger and more demographically diverse samples. Longitudinal designs may also help illuminate developmental changes in prosodic control and responsiveness to therapeutic interventions.

## Supplementary Material

Table S1

Figure S1

See the supplementary material for individual-level task performance metrics for children with DS and TD and individual pitch contours for DS participants across conditions.

## Figures and Tables

**TABLE I:** Individual Demographic Info for DS group

Participant ID	Age (Years & Months)	Age Equivalent Score (Years & Months)	Gender
DS01	8Y10M	2Y3M	M
DS02	13Y2M	2Y8M	F
DS03	15Y0M	2Y10M	M
DS05	12Y2M	7Y4M	M
DS06	13Y3M	2Y4M	F
DS07	8Y9M	3Y3M	F
DS09	15Y0M	2Y1M	F
DS10	11Y10M	<2Y0M	M
DS14	13Y2M	2Y9M	F

**TABLE II:** Mean F0 values and standard deviations for each group and condition

Group	Condition	Mean f0 (Hz)	SD
DS	given_nonshift	237.33	71.86
DS	given_shift	247.64	62.67
DS	new	227.65	57.86
TD	given_nonshift	287.30	66.09
TD	given_shift	296.93	63.61
TD	new	265.14	67.07

**TABLE III:** Linear Mixed-Effects Model Results for Mean F0

	Mean F0			
Predictors	Estimates		CI	p
(Intercept)	0.10	−0.20	0.40	0.516
group [TD]	−0.29	−0.56	−0.02	**0.034**
condition [given_shift]	0.03	−0.07	0.13	0.581
condition [new]	0.12	0.01	0.23	**0.031**
group [TD] x condition [given_shift]	0.03	−0.07	0.13	0.570
group [TD] x condition [new]	−0.08	−0.18	0.02	0.126
Random Effects				
σ^2^	0.44			
τ_00 Participants_	0.44			
τ_00 Item_	0.02			
ICC	0.51			
N _Participants_	31			
N _Item_	15			

Observations	593			
Marginal R^2^/Conditional R^2^	0.084/0.553			

**TABLE IV:** Mean intensity values and standard deviations for each group and condition

Group	Condition	Mean Intensity (dB)	SD
DS	given_nonshift	59.33	8.46
DS	given_shift	62.79	6.70
DS	new	58.86	6.98
TD	given_nonshift	48.20	9.15
TD	given_shift	48.79	8.71
TD	new	45.82	8.27

**TABLE V:** Linear Mixed-Effects Model Results for Mean Intensity

	Mean Intensity			
Predictors	Estimates		CI	p
(Intercept)	0.17	−0.11	0.45	0.224
group [TD]	0.63	0.37	0.89	**<0.001**
condition [given_shift]	−0.02	−0.10	0.06	0.634
condition [new]	0.16	0.08	0.24	**<0.001**
group [TD] x condition [given_shift]	−0.07	−0.14	0.01	0.083
group [TD] x condition [new]	0.04	−0.03	0.12	0.278
**Random Effects**				
σ^2^	0.24			
τ_00 Participants_	0.43			
τ_00 Item_	0.01			
ICC	0.65			
N _Participants_	31			
N _Item_	15			

Observations	593			
Marginal R^2^/Conditional R^2^	0.376/0.781			

**TABLE VI:** mean duration values and standard deviations for each group and condition

Group	Condition	Mean Duration (ms)	SD
DS	given_nonshift	531.21	162.96
DS	given_shift	617.55	237.12
DS	new	587.72	193.62
TD	given_nonshift	472.05	146.37
TD	given_shift	510.09	179.13
TD	new	591.46	189.58

**TABLE VII:** Linear Mixed-Effects Model Results for Duration

	Duration			
Predictors	Estimates		CI	p
(Intercept)	0.21	−0.23	0.66	0.338
group [TD]	0.14	−0.07	0.35	0.198
condition [given_shift]	−0.15	−0.28	−0.03	**0.016**
condition [new]	0.11	−0.02	0.24	0.091
group [TD] x condition [given_shift]	0.00	−0.12	0.12	0.986
group [TD] x condition [new]	0.13	0.01	0.25	**0.038**
**Random Effects**				
σ^2^	0.63			
τ_00 Participants_	0.24			
τ_00 Item_	0.36			
ICC	0.49			
N _Participants_	31			
N _Item_	15			

Observations	593			
Marginal R^2^/Conditional R^2^	0.018/0.498			

**TABLE VIII:** Summary of GAMM model fitted to the time-normalized f0 values (in semitones)

Parametric Coefficients				

Predictor	Estimate	Std. Error	*t*	Pr(>|t|)
(Intercept)	1.9101	0.7136	2.677	0.00745 **
condition(given_shift): group(DS)	0.6956	0.3895	1.786	0.07417 .
condition(new): group(DS)	−0.3831	0.2943	−1.302	0.19301
condition(given_nonshift): group(TD)	2.1701	0.8587	2.527	0.01152 *
condition(given_shift): group(TD)	2.5218	0.8634	2.921	0.00351 **
condition(new): group(TD)	1.1831	0.8366	1.414	0.15740

Smooth Terms				

Predictor	edf	Ref.df	*F*	*p*-value

s(scaled time): given_nonshift (DS)	1.000	1.000	0.002	0.96701
s(scaled time): given_shift (DS)	3.839	4.969	2.891	0.01345 *
s(scaled time): new (DS)	3.655	4.555	3.720	0.00303 **
s(scaled time): given_nonshift (TD)	4.876	6.203	2.182	0.04078 *
s(scaled time): given_shift (TD)	1.732	2.215	5.149	0.00504 **
s(scaled time): new (TD)	2.126	2.663	4.821	0.00562 **
s(scaled time, participant)	115.105	278.000	2.880	< 2e-16 ***

## Data Availability

The data that support the findings of this study are available from the corresponding author upon reasonable request.
