# Supplementary material for: Acoustic prominence: Understanding speech patterns in children with Down syndrome
Source: J Acoust Soc Am. Author manuscript; Available in PMC 2026 Mar 25. (PMC13014390; doi:10.1121/10.0039045)
Supplement: Table S1 [file NIHMS2152155-supplement-Table_S1.docx]

| **Group** | **Mean Trials Attempted** | **SE Trials Attempted** | **Min Trials Attempted** | **Max Trials Attempted** | **Mean Valid Trials** | **SE Valid Trials** | **Min Valid Trials** | **Max Valid Trials** |
| --- | --- | --- | --- | --- | --- | --- | --- | --- |
| DS | 35.00 | 3.89 | 9 | 48 | 27.44 | 3.40 | 5 | 35 |
| TD | 19.10 | 0.48 | 12 | 25 | 16.36 | 0.54 | 8 | 18 |

| **Participant** | **Group** | **Total Trials Attempted** | **Valid Trials Used** | **New Total** | **Given Total** | **New Correct** | **Given Correct** |
| --- | --- | --- | --- | --- | --- | --- | --- |
| DS01 | DS | 37 | 26 | 17 | 9 | 5 | 2 |
| DS02 | DS | 39 | 35 | 23 | 12 | 21 | 11 |
| DS03 | DS | 47 | 35 | 22 | 13 | 14 | 6 |
| DS05 | DS | 35 | 32 | 21 | 11 | 11 | 11 |
| DS06 | DS | 34 | 34 | 24 | 10 | 23 | 9 |
| DS07 | DS | 39 | 33 | 21 | 12 | 19 | 11 |
| DS09 | DS | 48 | 30 | 19 | 11 | 14 | 10 |
| DS10 | DS | 9 | 5 | 5 | 0 | 4 | 0 |
| DS14 | DS | 27 | 17 | 11 | 6 | 6 | 4 |
| TD01 | TD | 18 | 8 | 6 | 2 | 0 | 0 |
| TD02 | TD | 21 | 17 | 12 | 5 | 9 | 1 |
| TD03 | TD | 20 | 18 | 12 | 6 | 12 | 6 |
| TD04 | TD | 19 | 14 | 9 | 5 | 7 | 5 |
| TD05 | TD | 12 | 12 | 10 | 2 | 4 | 0 |
| TD06 | TD | 19 | 18 | 12 | 6 | 10 | 6 |
| TD08 | TD | 20 | 13 | 8 | 5 | 5 | 3 |
| TD10 | TD | 18 | 18 | 12 | 6 | 12 | 6 |
| TD12 | TD | 18 | 17 | 11 | 6 | 10 | 6 |
| TD13 | TD | 20 | 18 | 12 | 6 | 9 | 5 |
| TD14 | TD | 18 | 17 | 11 | 6 | 9 | 5 |
| TD15 | TD | 18 | 16 | 10 | 6 | 10 | 6 |
| TD16 | TD | 25 | 17 | 11 | 6 | 5 | 5 |
| TD19 | TD | 19 | 18 | 12 | 6 | 11 | 5 |
| TD20 | TD | 18 | 18 | 12 | 6 | 12 | 6 |
| TD21 | TD | 21 | 18 | 12 | 6 | 12 | 6 |
| TD22 | TD | 19 | 17 | 11 | 6 | 11 | 5 |
| TD23 | TD | 19 | 16 | 11 | 5 | 8 | 4 |
| TD24 | TD | 20 | 17 | 11 | 6 | 9 | 5 |
| TD25 | TD | 18 | 18 | 13 | 5 | 13 | 5 |
| TD26 | TD | 20 | 18 | 12 | 6 | 8 | 4 |
| TD27 | TD | 20 | 17 | 10 | 7 | 8 | 6 |

Table S1. Individual-Level Task Performance Metrics for Children with DS and TD
