# Supplementary figures and images for: Acoustic prominence: Understanding speech patterns in children with Down syndrome
Source: J Acoust Soc Am. Author manuscript; Available in PMC 2026 Mar 25. (PMC13014390; doi:10.1121/10.0039045)

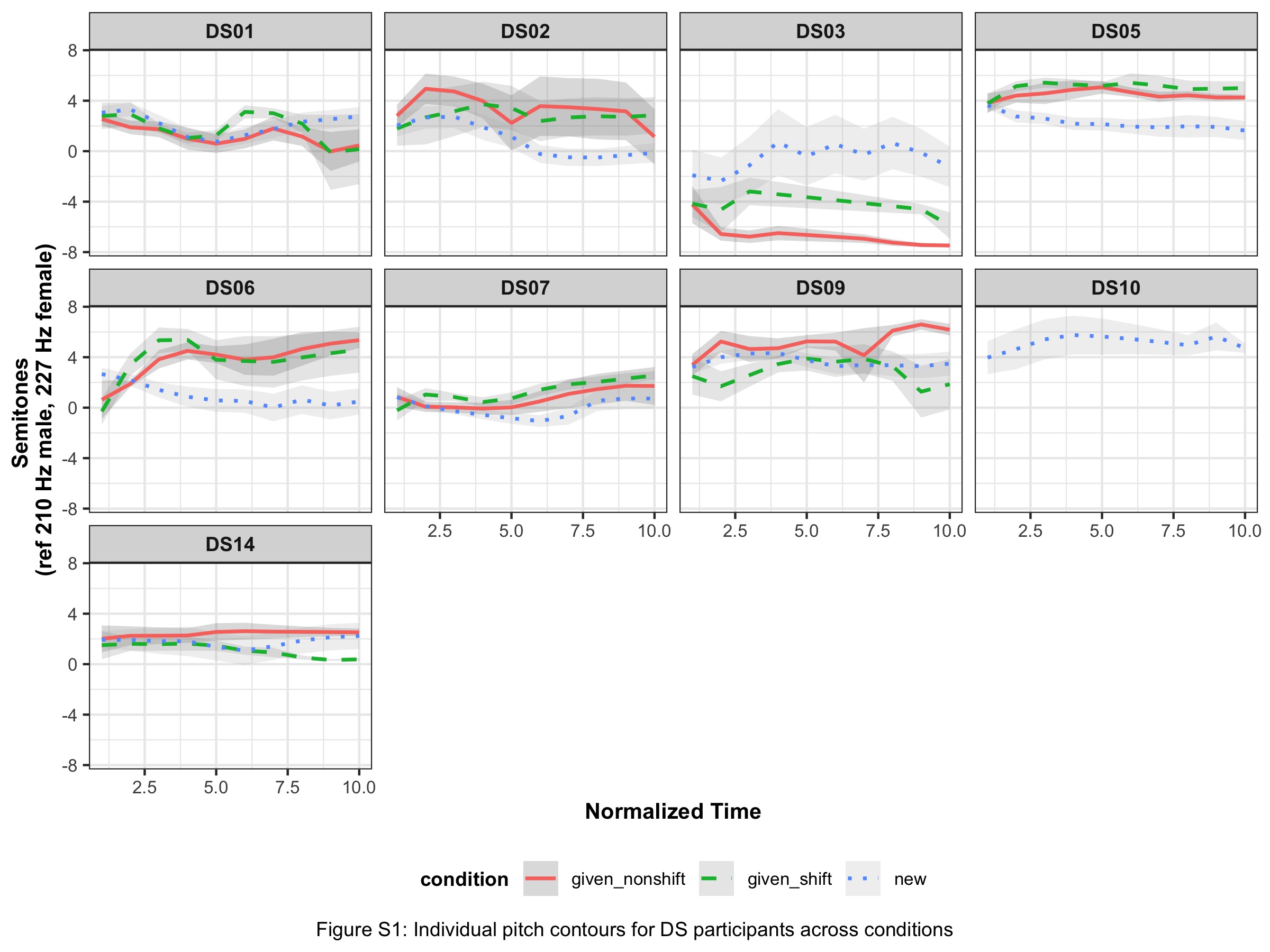

Supplement: Figure S1 [file NIHMS2152155-supplement-Figure_S1.jpeg]
